# Blood transcriptome profile induced by an efficacious vaccine formulated with salivary antigens from cattle ticks

**DOI:** 10.1038/s41541-019-0145-1

**Published:** 2019-12-18

**Authors:** Sandra R. Maruyama, Benilton Carvalho, Mar González-Porta, Johan Rung, Alvis Brazma, Luiz Gustavo Gardinassi, Beatriz R. Ferreira, Tamy M. Banin, Cecília J. Veríssimo, Luciana M. Katiki, Isabel K. F. de Miranda-Santos

**Affiliations:** 1 Ribeirão Preto School of Medicine, University of São Paulo, Ribeirão Preto, SP Brazil; 2 University of Campinas, Campinas, SP Brazil; 3 European Molecular Biology Laboratory, European Bioinformatics Institute, EMBL-EBI, Hinxton, UK; 4 School of Pharmaceutical Sciences of Ribeirão Preto, University of São Paulo, Ribeirão Preto, SP Brazil; 5 Ribeirão Preto School of Nursing, University of São Paulo, Ribeirão Preto, SP Brazil; 6 São Paulo Institute of Animal Science–IZ, Nova Odessa, SP Brazil; 7 Present Address: Department of Genetics and Evolution, Center for Biological Sciences and Health, Federal University of São Carlos, São Carlos, SP Brazil; 8 Present Address: Illumina Centre, Cambridge, UK; 9 Present Address: Department of Immunology, Genetics and Pathology, Uppsala University, Uppsala, Sweden

**Keywords:** Predictive markers, Protein vaccines, Protein vaccines

## Abstract

Ticks cause massive damage to livestock and vaccines are one sustainable alternative for the acaricide poisons currently heavily used to control infestations. An experimental vaccine adjuvanted with alum and composed by four recombinant salivary antigens mined with reverse vaccinology from a transcriptome of salivary glands from *Rhipicephalus microplus* ticks was previously shown to present an overall efficacy of 73.2% and cause a significant decrease of tick loads in artificially tick-infested, immunized heifers; this decrease was accompanied by increased levels of antigen-specific IgG1 and IgG2 antibodies, which were boosted during a challenge infestation. In order to gain insights into the systemic effects induced by the vaccine and by the tick challenge we now report the gene expression profile of these hosts’ whole-blood leukocytes with RNA-seq followed by functional analyses. These analyses show that vaccination induced unique responses to infestations; genes upregulated in the comparisons were enriched for processes associated with chemotaxis, cell adhesion, T-cell responses and wound repair. Blood transcriptional modules were enriched for activation of dendritic cells, cell cycle, phosphatidylinositol signaling, and platelets. Together, the results indicate that by neutralizing the tick’s salivary mediators of parasitism with vaccine-induced antibodies, the bovine host is able to mount normal homeostatic responses that hinder tick attachment and haematophagy and that the tick otherwise suppresses with its saliva.

## Tick vaccines: multivalent formulation activates leukocytes systemically

Available vaccines to protect against the bovine tick *Rhipicephalus microplus* have variable efficacy, but a new experimental vaccine formulation featuring four *R. microplus* saliva antigens has shown enhanced protection against tick infestation by triggering the production of antigen-specific IgG antibodies in vaccinated bovines. Now, Isabel de Miranda-Santos and colleagues performed RNA sequencing analysis of peripheral blood immune cells from vaccinated calves and show that this multivalent vaccine induces systemic activation of leukocytes that prevents effective attachment of *R. microplus* ticks. The researchers report an increase in the expression of genes associated with chemotaxis, cell adhesion, T-cell responses and wound repair, and find evidence of enhanced activation of dendritic cells and platelets. The genes and biological pathways identified underline the importance of using vaccines to neutralize tick saliva, freeing systemic immune responses and skin immunity to prevent tick infestation in farm animals.

## Introduction

*Rhipicephalus microplus*, the cattle tick, causes heavy infestations in taurine breeds of cattle, severely affecting their health and causing huge production losses where they are employed.^1^ Vaccines represent an alternative to acaricides to control tick infestations. TickGard^2^ and GAVAC^3^ are the vaccines to control cattle ticks that went to market. Both are based on a single antigen, the tick gut glycoprotein Bm86. The efficacy of both vaccines is variable^4–7^ and since the antigen is hidden from the hosts immune system during parasitism, the memory they induce is short-lived, thus requiring at least one boost a year,^8,9^ which makes them unattractive to producers. An approach to obtain a level of vaccine efficacy compatible with ease of management and good production in the field consists of formulating a multicomponent vaccine combining many antigens, in an attempt to decrease the proportion of poor responders to the vaccine and to also target the tick’s multiple mediators of parasitism. In addition, since these mediators are mostly in the saliva that ticks inoculate in their hosts, memory may be boosted when cattle are naturally exposed to ticks.

In a previous study, we tested an experimental vaccine formulation composed by four recombinant salivary antigens from *R. microplus*, named Rm39, Rm76, Rm180, Rm239, which were predicted in silico as a glycine-rich cement protein, an immunoglobulin binding-protein, a thrombin inhibitor, and a metalloprotease, respectively. These antigens were chosen due to their roles in parasitism of the host: attachment of the tick (Rm39), abrogation of antibody responses (Rm76), anticoagulation (Rm180) and disruption of the extracellular matrix to create the blood-feeding pool (Rm239). The vaccine caused a significant decrease of tick loads in artificially tick-infested, immunized heifers; this decrease was accompanied by increased levels of antigen-specific IgG1 and IgG2 antibodies, including during a challenge infestation.^10^ In order to gain insight into the mechanisms that must be induced by an efficacious vaccine and to identify potential biomarkers of efficacy, we examined the transcriptional profile elicited in blood leukocytes by this multicomponent tick vaccine. For this, we performed an RNA-seq analysis of blood cells from vaccinated and control animals of a genetically tick-susceptible breed, the target for such a vaccine. The present study presents a bovine transcriptome sequencing data obtained in the context of Systems Vaccinology^11,12^ applied to understanding effector mechanisms of immunological-based control of cattle ticks.

## Results

### Characteristics of the blood transcriptome and most significant differentially expressed genes

**Fig. 1:**
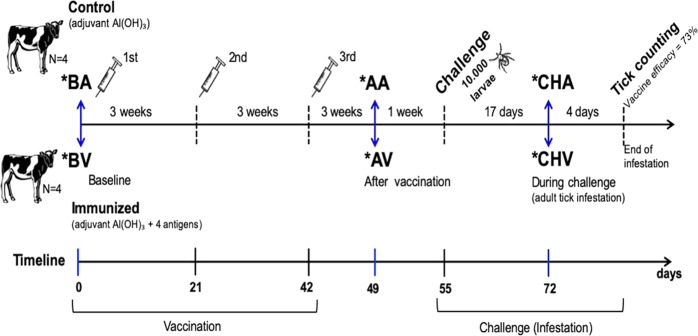
Experimental design for obtaining blood transcriptome. Schematic representation of vaccination trial indicating the sample collection periods (*) used for blood transcriptome profiling by RNA-seq. Female Holstein calves (*n* = 4) were immunized by intramuscular route with four recombinant salivary antigens of *Rhiphicephalus microplus* adjuvanted with aluminium hydroxide [Al(OH)_3_, prepared in separate injections] three times with 3-weeks intervals or injected only with saline and adjuvant (control group). Blood samples for the RNA-seq experiment were collected before vaccination (BV) or administration of control adjuvant (BA), after vaccination (AV) or administration of control adjuvant (AA), and after challenge tick infestation in vaccinated (CHV) and control adjuvant (CHA) animals. Adapted from Maruyama et al.^10^

Details of the immunization scheme and challenge with ticks are reported in our previous study^10^ and depicted in Fig. 1. Blood samples from that experiment were employed in this study to perform RNA-seq analysis; 24 blood samples were collected from calves in the control (adjuvant only) and vaccinated groups (*n* = 4 in each group) at three different time points: before vaccination (BA, adjuvant only group; BV, vaccinated group), on day 7 after the third and last vaccine dose (AA, adjuvant only group; AV, vaccinated group) and on day 17 after a challenge with tick larvae, when they have molted to adults and the heifers have thus been exposed to most of the parasite’s salivary antigens, including those in the vaccine (CHA, adjuvant only group; CHV, vaccinated group).

The 24 Illumina RNA-seq libraries were sequenced in four lanes with an average of 31 million single-end reads and 3.1 Gb per library. After quality assessment of the libraries, the reads were mapped to the bovine genome and quantified at the gene level. Subsequently, the differentially expressed genes (DEGs) across the experimental conditions were determined. In total 13,952 genes were expressed across all 24 samples, presenting a low biological coefficient of variation (BCV = 16.2%) among the biological replicates.

The DEGs were calculated in a comparative analyses to answer the following questions: (a) which genes respond to vaccination (i.e. after vaccination [AV] vs. before vaccination [BV]) and to infestation (challenged animals that received adjuvant only [CHA] vs. the same animals before they received adjuvant [BA]); (b) what are the interactions between vaccination and infestation, i.e. which genes are differentially expressed in vaccinated, infested animals (challenged, vaccinated animals [CHV] vs. the same animals before vaccination [BV] and challenged, vaccinated animals [CHV] vs. challenged animals that received adjuvant only [CHA]). For each comparison, we observed several differentially expressed genes using an FDR (false discovery rate) cut off of <0.05, as follows: (a) AV vs. BV: 424 (217 up- and 207 downregulated); (b) CHA vs. BA: 2,071 (1285 up- and 786 downregulated); (c) CHV vs. BV: 171 (97 up- and 74 downregulated) and CHV vs. CHA: 74 (37 up- and 37 downregulated) at FDR < 0.1. The top ten most significant DEGs identified are listed in Table 1. Some of these DEGs, such as TIEG2 (Krueppel-like factor 11) and BT.64205 (antigen WC1.1 precursor, also named BoWC1.1, WC1 isolate CH149, CD163 molecule-like 1), were differentially expressed in two or more comparisons. Many uncharacterised proteins were highly differentially expressed. Other possible comparisons were also performed: BA vs AA, CHA vs AA and CHV vs AV, resulting in 985, 79 and 70 differentially expressed genes, respectively. All DEGs are described in ‘Supplementary Data 1, sheets a-dDEG’.

**Table 1:** Description of genes found to be most significantly differentially expressed in AV, CHV and CHV vs.

Bovine gene ID	Gene name	Description^a^	FDR^b^	logFC^b, c^
Effect of vaccination → Comparison: [AV vs. BV] vs. [AA vs. BA]); 424 DEGs				
ENSBTAG00000014069	BT.102008	Uncharacterised protein	3.68E-29	−2.35
ENSBTAG00000046218	TIEG2	Krueppel-like factor 11	5.53E-16	−1.60
ENSBTAG00000021008	ZNF219	Uncharacterised protein	2.01E-10	1.55
ENSBTAG00000039817	BT.66792	Uncharacterised protein	1.09E-08	1.56
ENSBTAG00000026531	BT.64205	Antigen WC1.1 precursor	1.38E-08	1.59
ENSBTAG00000039256		Uncharacterised protein	2.07E-08	1.71
ENSBTAG00000047302		Uncharacterised protein	2.26E-08	−2.84
ENSBTAG00000024318	BT.25501	Uncharacterised protein	4.99E-08	1.62
ENSBTAG00000008132	SOX13	Uncharacterised protein	8.68E-08	1.47
ENSBTAG00000039470	WC1	Uncharacterised protein LOC751809 precursor	1.22E-07	1.54
Effect of infestation only → Comparison: CHA vs. BA; 2.071 DEGs				
ENSBTAG00000037578	BT.62519	Uncharacterised protein	1.20E-08	−1.26
ENSBTAG00000021437	GPR174	Uncharacterised protein	3.04E-08	2.07
ENSBTAG00000015310	SLC25A36	Solute carrier family 25 member 36	1.94E-07	1.39
ENSBTAG00000038337	PACAP	Plasma cell-induced resident endoplasmic reticulum protein	1.94E-07	−1.78
ENSBTAG00000046218	TIEG2	Krueppel-like factor 11	6.55E-06	−1.03
ENSBTAG00000047874	PRPF38B	Uncharacterised protein	6.54e-06	0.78
ENSBTAG00000020355	KLF4	Krueppel-like factor 4	7.07e-06	0.89
ENSBTAG00000046322		Uncharacterised protein	9.84e-06	−1.29
ENSBTAG00000046959	BT.85663	CGG triplet repeat-binding protein 1	9.84e-06	0.85
ENSBTAG00000000223	PPM1B	Protein phosphatase 1B	9.84e-06	0.78
Infestation under effect of vaccination → Comparison: [CHV vs. BV] vs. [CHA vs. BA]; 171 DEGs				
ENSBTAG00000014069	BT.102008	Uncharacterised protein	4.19E-07	−1.24
ENSBTAG00000046218	TIEG2	Krueppel-like factor 11	1.40E-05	−1.03
ENSBTAG00000008132	SOX13	Uncharacterised protein	2.23E-03	1.24
ENSBTAG00000002096	FCGR3A	Low-affinity immunoglobulin γFc region receptor III 1	2.37E-03	−1.32
ENSBTAG00000026531	BT.64205	Antigen WC1.1 precursor	2.37E-03	1.25
ENSBTAG00000037578	BT.62519	Uncharacterised protein	3.4E-03	−0.89
ENSBTAG00000018828	ATN1	Atrophin-1	4.0E-03	0.69
ENSBTAG00000039470	WC1	Uncharacterised protein LOC751809 precursor	4.0E-03	1.23
ENSBTAG00000017670	BT.60918	Interferon-induced guanylate-binding protein 1	5.3E-03	−1.11
ENSBTAG00000001725	CXCL10	C-X-C motif chemokine 10	5.3E-03	−1.25
Infestation under effect of vaccination → Comparison: CHV vs. CHA; 74 DEGs				
ENSBTAG00000034295	BT.18959	Lymphocyte activation gene 3 protein precursor	2.08E-03	1.25
ENSBTAG00000008132	SOX13	Uncharacterised protein	2.08E-03	1.74
ENSBTAG00000026531	BT.64205	Antigen WC1.1 precursor	2.08E-03	1.79
ENSBTAG00000039817	BT.66792	Uncharacterised protein	2.08E-03	1.73
ENSBTAG00000004136	NFE2L3	Nuclear factor erythroid 2-related factor 3	7.27E-03	−1.01
ENSBTAG00000039470	WC1	Uncharacterised protein LOC751809 precursor	8.7E-03	1.70
ENSBTAG00000024318	BT.25501	Uncharacterised protein	1.0E-03	1.72
ENSBTAG00000039847		Uncharacterised protein	1.8E-03	1.53
ENSBTAG00000039256		Uncharacterised protein	3.4E-03	1.68
ENSBTAG00000022244		Uncharacterised protein	3.4E-03	1.75

CHA comparisons—the top ten most significantly (based on FDR value) differentially expressed genes (DEGs) affected by vaccination and/or tick infestation

FDR false discovery rate (adjusted p-value)

^a^gene description obtained from BioMart-Ensembl database

^b^Read counts for calculation

^c^Fold change (log transformation) values of expression for: vaccinated group after vaccination; control group after infestation; vaccinated group after challenge with ticks

### Profiles of DEGs according to the experimental conditions

**Fig. 2:**
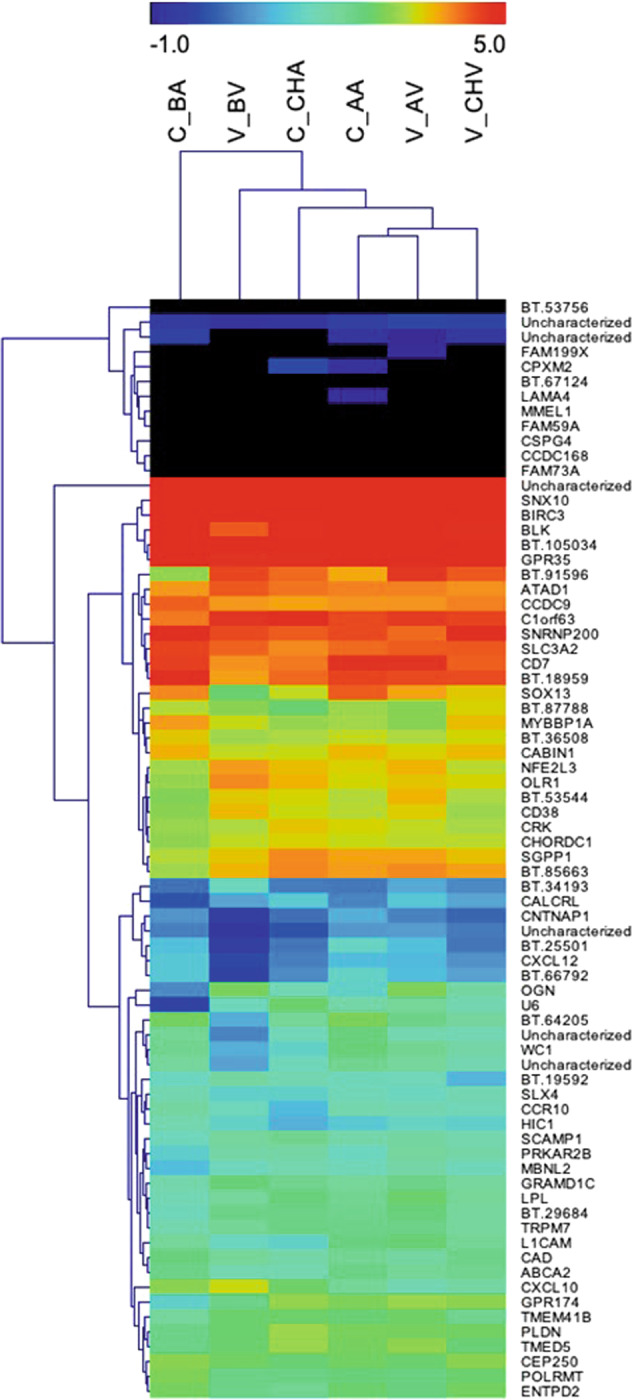
Exploratory data analyses of blood transcriptional profiling in Holstein Heifers upon experimental vaccination against ticks. Hierarchical clustering of differentially expressed genes (DEGs) in infested vaccinated animals, CHV vs. CHA pairwise comparison (74 DEGs): The colour scale represents log_2_RPKM, in which the transition of colours from blue to red indicates increasing gene expression levels. The heatmap show the levels of gene expression through RPKM values of DEGs. The colour transition from black towards rainbow variation means low to high expression level. The black colour indicates genes with expression at log_2_RPKM < −1.0 (very low levels), and red indicates genes with expression log_2_RPKM ≥ 5.0 (very high levels). BV before vaccination, BA before adjuvant, AV after vaccination, AA after adjuvant, CHV vaccinated animals during challenge (tick infestation) and CHA control animals during challenge (tick infestation).

To explore how the patterns of DEGs are associated across the groups during different time points of the experiment, hierarchical clustering (HCL) analyses were performed and the results were plotted as a heatmap (Fig. 2). To this end, RPKM values of the DEG lists were obtained through the normalisation of read counts according to gene length. Thus, we compared the levels of gene expression between genes. The results obtained in the heatmap showed that the expression profile of DEGs clustered according to the experimental conditions. Interestingly, the 74 DEGs identified in CHV vs. CHA pairwise comparisons presented an expression profile in which the infested vaccinated group (CHV) was clustered in an isolated position in the heatmap (Fig. 2 right group). These HCL analyses suggest that the multicomponent vaccine induced a unique transcriptional profile regulation of blood leukocytes from bovines responding to tick infestations. The identified DEGs will be useful for the elucidation of the molecular mechanisms involved in the response induced in vaccinated animals that reduces tick infestations.

### Functional analyses of transcriptome

To identify the biological pathways, networks and GO (Gene Ontology) processes associated with the genes affected after vaccination and tick infestation, the MetaCore™ platform for functional genomics data from Clarivate Analytics was used. The bovine gene IDs were converted in orthologous human gene IDs using BioMart.^13^ Approximately 90% of bovine genes (from DEG lists) demonstrated homology with human genes. From the DEG list of AV, CHV and CHA vs. CHV comparisons, 380, 152 and 71 human orthologues, respectively, were retrieved from BioMart. The functional analyses of the genes affected after vaccination and tick infestation were enriched for several biological functions associated with immune responses (AV comparisons, CHV comparisons and CHV vs. CHA comparisons, Tables 2–4, respectively). The genes upregulated in the three comparisons were enriched for processes associated with chemotaxis, cell adhesion, the T-cell immune response, and organ regeneration, this latter process most likely associated with wound healing of the skin. The downregulated genes were enriched in other categories of immune processes, such as inflammation through the complement system and interferon signalling, and cell cycle processes.

**Table 2:** Enrichment analyses performed with Metacore in AV comparisons.

Pathway Map; FDR^a^	Network; FDR^a^	GO Process; FDR^a^
Animals after vaccination (AV): upregulated genes		
Development_SDF-1 signalling in hematopoietic stem cell homing; 0.028	Cell adhesion_Attractive and repulsive receptors; 0.010	Regulation of cell migration; 6.12E-06
Regulation of lipid metabolism_Regulation of fatty acid synthase activity in hepatocytes; 0.028	Development_Regulation of angiogenesis; 0.010	Regulation of cell motility; 6.12E-06
Development_Oligodendrocyte differentiation from adult stem cells; 0.028	Development_Neurogenesis_Axonal guidance; 0.010	Locomotion; 6.12E-06
Cell adhesion_ECM remodelling; 0.028	Cell adhesion_Amyloid proteins; 0.010	Regulation of locomotion; 6.12E-06
ENaC regulation in normal and CF airways; 0.028	Development_Neurogenesis in general; 0.035	Regulation of cellular component movement; 6.12E-06
Immune response_T cell receptor signalling pathway; 0.028	Cell adhesion_Leucocyte chemotaxis; 0.041	Axon guidance; 6.12E-06
G-protein signalling_M-RAS regulation pathway; 0.028		Neuron projection guidance; 6.12E-06
G-protein signaling_TC21 regulation pathway 0.028		Chemotaxis; 6.12E-06
Resolution of inflammation in healing myocardial infarction; 0.028		Cellular component movement; 6.12E-06
Animals after vaccination (AV): downregulated genes		
Cell cycle_Chromosome condensation in prometaphase; 1.26E-08	Cell cycle_Mitosis; 3.60E-04	Mitotic cell cycle; 8.70E-14
Cell cycle_Role of APC in cell cycle regulation; 1.94E-07	Cell cycle_Core; 9.57E-04	Mitotic cell cycle process; 3.03E-12
Cell cycle_Role of Nek in cell cycle regulation; 5.60E-06	Cell cycle_G2-M; 0.012	Mitotic nuclear division; 5.30E-10
Cell cycle_Initiation of mitosis; 4.70E-05	Cytoskeleton_Spindle microtubules; 0.014	Cell cycle; 9.40E-10
Cell cycle_Spindle assembly and chromosome separation; 1.44E-04	Cell cycle_S phase; 0.015	Eating behaviour; 2.01E-09
Cell cycle_The metaphase checkpoint; 2.04E-04	Proteolysis_Ubiquitin-proteasomal proteolysis; 0.026	Cell cycle process; 4.47E-09
Cell cycle_Sister chromatid cohesion; 7.30E-04		Response to stress; 9.71E-09
Reproduction_Progesterone-mediated oocyte maturation; 4.25E-03		Nuclear division; 1.05E-07
Cell cycle_Nucleocytoplasmic transport of CDK/Cyclins; 5.70E-03		Response to other organism; 1.59E-07

^a^Only biological processes enriched with FDR < 0.05 are listed

**Table 3:** Enrichment analyses performed with Metacore in CHV comparisons.

Pathway Map; FDR^a^	Network; FDR^a^	GO Process; FDR^a^
Vaccinated animals during challenge [tick infestation (CHV)]: upregulated genes		
Cytoskeleton remodelling_Cytoskeleton remodelling; 4.64E-03		Anatomical structure development; 2.83E-07
Regulation of lipid metabolism_Regulation of fatty acid synthase activity in hepatocytes; 4.64E-03		Developmental process; 2.83E-07
Development_Role of IL-8 in angiogenesis; 4.34E-03		Single-organism developmental process; 5.10E-07
Regulation of lipid metabolism_Regulation of lipid metabolism via LXR, NF-Y and SREBP; 4.91E-02		Positive regulation of biological process; 5.10E-07
Cell adhesion_Histamine H1 receptor signalling in the interruption of cell barrier integrity; 2.52E-02		Multicellular organismal development; 7.42E-07
Cell adhesion_Integrin-mediated cell adhesion and migration; 2.53E-02		System development; 7.42E-07
Cytoskeleton remodelling_Substance P mediated membrane blebbing; 4.13E-02		Myeloid cell differentiation; 3.83E-06
Transcription_Sirtuin6 regulation and functions; 4.37E-02		Heamatopoietic or lymphoid organ development; 1.31E-05
Vaccinated animals during challenge [tick infestation (CHV)]: downregulated genes		
Immune response_Classical complement pathway; 8.79E-04	Inflammation_Complement system; 3.41E-04	Immune response; 2.74E-09
Protein folding and maturation_Angiotensin system maturation\Human version; 6.03E-03	Inflammation_Interferon signalling; 1.84E-02	Response to stress; 1.48E-08
Protein folding and maturation_Angiotensin system maturation\Rodent version; 6.03E-03		Immune system process; 1.48E-08
Immune response_Lectin induced complement pathway; 6.03E-03		Defence response; 1.55E-08
Glycolysis and gluconeogenesis p.3 / Human version; 2.42E-02		Innate immune response; 5.50E-08
		Defence response to protozoan; 2.29E-07
		Response to interferon-beta; 2.07E-05

^a^Only biological processes enriched with FDR < 0.05 are listed

**Table 4:** Enrichment analyses performed with Metacore in CHV vs. CHA comparisons.

Pathway Map; FDR^a^	Network; FDR^a^	GO Process; FDR^a^
CHA vs. CHV: upregulated genes		
Langerhans cell migration to lymph nodes in allergic contact dermatitis; 0.042	Chemotaxis; 0.054	Chemokine-mediated signalling pathway; 0.03
Immune response_T cell subsets: cell surface markers; 0.046		Positive regulation of response to stimulus; 0.03
CHA vs. CHV: downregulated genes		
Development_PDGF signalling via MAPK cascades; 6.61E-05	Apoptosis_Anti-Apoptosis mediated by external signals via MAPK and JAK/STAT; 4.35E-03	Response to antipsychotic drug; 3.37E-06
Transport_The role of AVP in regulation of Aquaporin 2 and renal water reabsorption; 7.96E-05	Apoptosis_Anti-apoptosis mediated by external signals via NF-kB; 5.05E-03	Positive regulation of phospholipase C activity; 3.37E-06
Development_Non-genomic action of Retinoic acid in cell differentiation; 1.11E-04	Apoptosis_Anti-Apoptosis mediated by external signals via PI3K/AKT; 5.05E-03	Regulation of phospholipase C activity; 3.37E-06
Cytoskeleton remodelling_Role of PDGFs in cell migration; 7.60E-04	Reproduction_Spermatogenesis. Motility and copulation; 0.04	Positive regulation of phospholipase activity; 8.68E-06
Role of Diethylhexyl Phthalate and Tributyltin in fat cell differentiation; 1.11E-03		Positive regulation of lipase activity; 1.12E-05
Development_PDGF signalling via STATs and NF-kB; 1.35E-03		Regulation of phospholipase activity; 1.12E-05
Normal and pathological TGF-beta-mediated regulation of cell proliferation; 1.44E-03		Response to clozapine; 1.30E-05
Development_Beta adrenergic receptors in brown adipocyte differentiation; 1.81E-03		Regulation of lipase activity; 5.97E-05
CFTR-dependent regulation of ion channels in CF; 2.22E-03		Immune system process; 7.49E-05
wtCFTR and deltaF508 traffic/Membrane Expression (normal and CF); 2.78E-03		negative regulation of cAMP-Dependent protein kinase activity; 7.49E-05

^a^Only biological processes enriched with FDR < 0.05 are listed

**Fig. 3:**
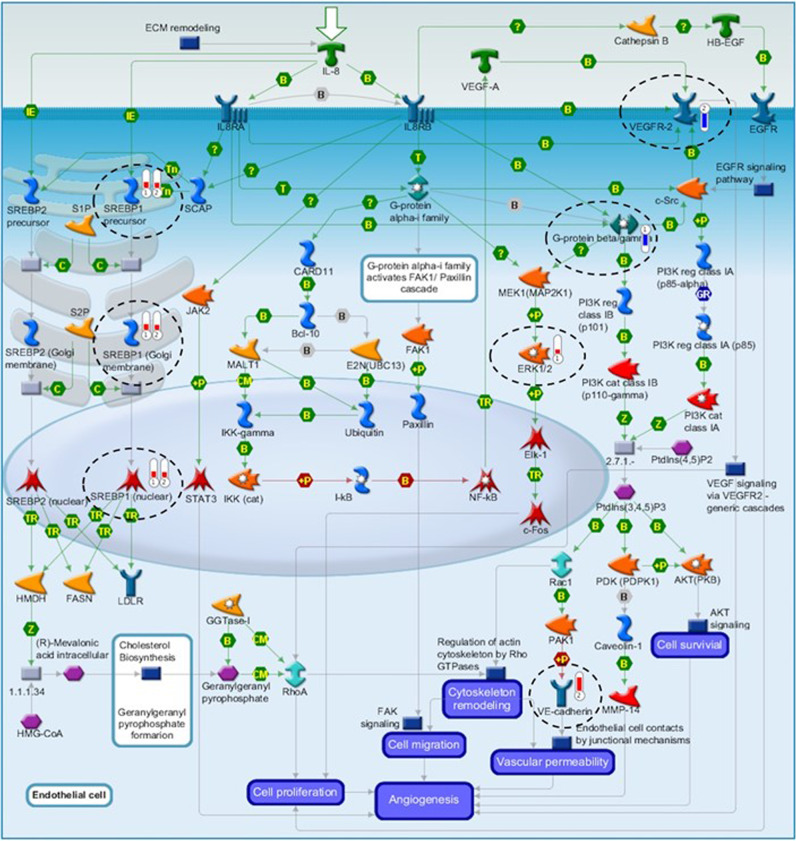
Metacore Pathway obtained with DEGs in blood from vaccinated calves. The pathway map “Role of IL-8 angiogenesis” obtained by functional analysis of differentially expressed genes in vaccinated calves: Thermometers 1 and 2 next to gene/name pictograms depict the genes present in AV and CHV comparisons, respectively, which are highlighted with opened dashed circles. The red thermometer indicates upregulated genes and the blue thermometer indicates downregulated genes; the thermometer level represents the intensity of the log_2_fold-change. Image generated using Metacore.

Regarding pathway analysis in Metacore outputs, the pathway maps are graphic images drawn to represent biochemical pathways or signalling cascades; the genes in the dataset that are present in the given pathway map are indicated in a thermometer pictogram that is either in blue (negative logFC, downregulated gene) or red (positive logFC, upregulated gene). The biological pathway “Role of IL-8 angiogenesis” (Fig. 3) was highly enriched in the CHV comparison.

**Fig. 4:**
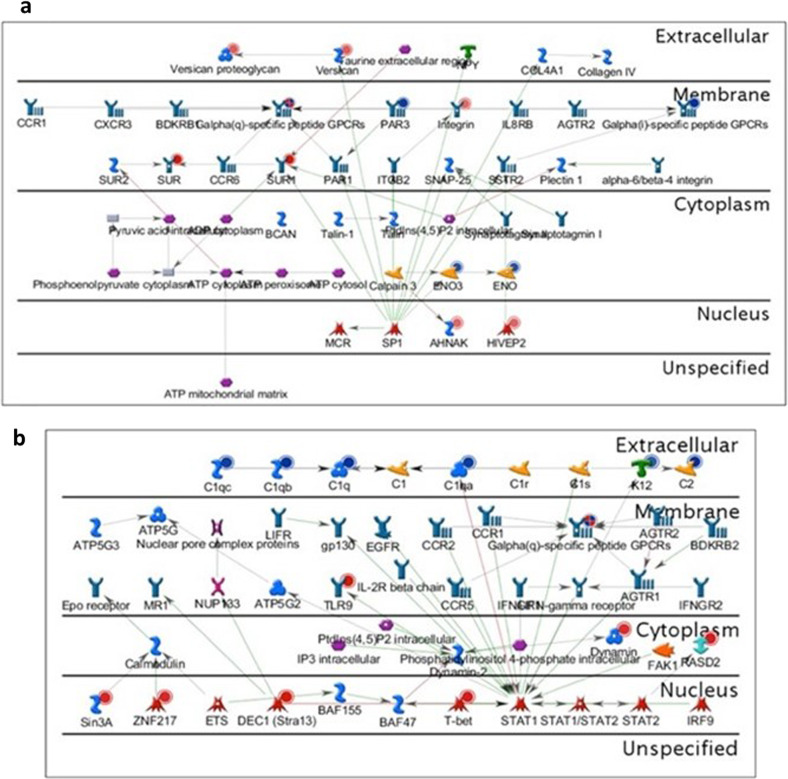
Metacore Network obtained with DEGs in blood from vaccinated calves. Network statistics analysis of DEGs from AV and CHV comparisons generated using Metacore: genes present in the dataset are indicated with circles: red circles indicate upregulated genes, blue circles indicate downregulated genes and mixed circles indicate mixed gene expression. The grade of colour inside the circle represents the intensity of the log_2_FC values, accordingly. **a** Network names (percentage of genes involved in each): SUR1, Versican, ENO3, AHNAK, HIVEP2 [related processes: cellular component movement (52.8%), locomotion (50.0%), response to wounding (50.0%), chemokine-mediated signalling pathway (16.7%), leukocyte chemotaxis (22.2%); *P* = 1.59e-18]. **b** Network names: DEC1 (Stra13). C2, TLR9, C1qa, RASD2 [related processes: regulation of immune response (53.2%), immune system process (70.2%), immune response (57.4%), regulation of immune system process (55.3%), defence response (57.4%); *P* = 9.02e-30].

Another functional insight comes from the outputs generated by Metacore for networks, which comprise graphical representations displaying objects (molecular entities) connected with other molecules in reactions, processes or relationships, each having a number of biological attributes with symbolic representations in the outputs. The “Network Statistics” feature in Metacore calculates the most prevalent networks involved in a given dataset, combining objects from different networks in a unique chart. Network objects (genes) presented in the dataset (AV and CHV comparisons) are indicated as either a blue (negative logFC, downregulated gene), red (positive logFC, upregulated gene) or mixed-coloured (when positive and negative values are present for the same object) circle. The two most significant network compilation images were generated through the “Network Statistics” analysis using the DEGs from AV and CHV comparison-enriched processes; these networks pertained to regulation of the immune response, response to wounding and leukocyte chemotaxis (Fig. 4a, b).

### Evaluation of simultaneous increases and decreases of expression of sets of genes

**Fig. 5:**
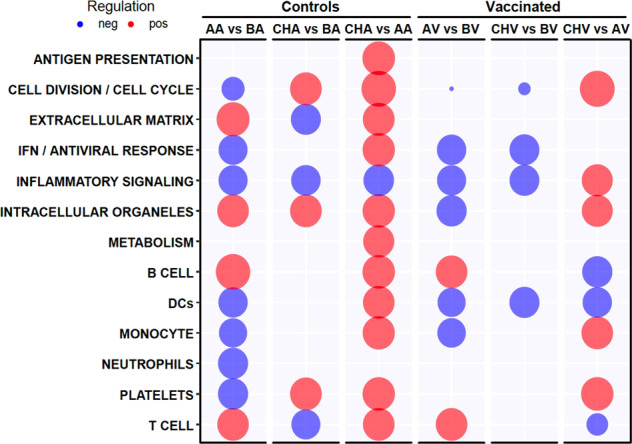
Blood transcription module (BTM) analysis of vaccination and tick challenge. Gene set enrichment analysis was conducted to identify BTMs associated with the response to adjuvant, to vaccination or to tick challenge. BTMs were grouped into high-level annotation processes or cell lineages (for detailed results see ‘Supplementary Data 1, sheet eBTMs’). Log counts per million (CPM) values were used for comparisons with baseline levels. Baseline-normalized log CPM values were used for comparisons between tick challenge and adjuvant injection or vaccination. Bubble sizes indicate the sum of normalized enrichment scores (NES) for high-level annotation BMTs. Red indicates positive association and blue indicates negative association. Only BTMs enriched with a nominal *p*-value < 0.05 and false discovery rate (FDR) < 0.1 were included.

To gain further insights about the transcriptional responses to vaccination and tick challenge, we performed gene set enrichment analysis (GSEA) using the framework of Blood Transcription Modules (BTM) as gene sets.^14^ GSEA accounts for simultaneously increased and decreased expression of a set of genes in a given module, the members of which may not have been identified as differentially expressed at the level of single-gene analysis. Over 90 BTMs were significantly associated with the entire dataset for at least one statistical comparison (‘Supplementary Data 1, sheet eBTMs’). BTMs were grouped into high-level annotation based on common pathways or cell lineages to facilitate the interpretation and visualization (Fig. 5). Compared to baseline gene expression, adjuvant injection induced higher expression of genes related to extracellular matrix, intracellular organelles, B cells and T cells, while downregulated genes were related to cell cycle and division, type I interferon response, inflammatory signaling, dendritic cells (DCs), monocytes, neutrophils and platelets. Vaccination also induced higher expression of genes related to B and T cells, but not extracellular matrix. Still compared to baseline levels, challenge of control group induced higher expression of genes associated with cell cycle and division, intracellular organelles and platelets, whereas genes related to T cells were downregulated. Genes from vaccinated and challenged animals were only negatively associated with high-level annotation BTMs when compared to baseline levels. These include BTMs related to cell cycle and division, type I interferon, inflammatory signaling and DCs. Of note, comparisons between experimental groups, CHA vs AA or CHV vs AV, using log CPM values resulted in only seven significant associations with BTMs. The lack of statistical significance in GSEA suggested high individual variability in gene expression between animals composing the same experimental groups. Therefore, to remove individual confounding effects, we used baseline-subtracted log CPM values to perform GSEA, as previously described^15^ Tick challenge after adjuvant injection induced positive associations with most high-level annotation BTMs, while genes related to inflammatory signaling were downregulated. Furthermore, tick challenge after vaccination induced higher expression of genes related to cell cycle and division, inflammatory signaling, intracellular organelles, monocytes and platelets, while negative associations included BTMs for DCs, B and T cells. The list of all BTM with their gene compositions can be downloaded from ‘Supplementary Data 1, sheet eBTMs’.

## Discussion

The RNA-seq analysis of whole blood from vaccinated calves indicated sets of genes that were differentially expressed and likely involved in the molecular mechanisms elicited during vaccination and tick infestation. Several genes associated with immune responses were differentially expressed in vaccinated calves. The transcriptional profile of 74 DEGs from the CHV vs. CHA comparison showed the efficient segregation of the vaccinated group during tick infestation, suggesting that this set of genes represents a transcriptional signature for protective vaccination against ticks.^10^ Several genes presented immune-related functions, such as WC1, CD7, BLK, CXCL10, CXCL12 and CCR10. They are functionally highlighted and discussed below (Fig. 2).

The bovine genome contains 13 members of the WC1 gene family, organised into two loci on chromosome 5.^16^ Two of these members (ENSBTAG00000039470 and ENSBTAG00000026531) were significantly upregulated (FDR < 0.01) in the CHV group. The WC1 gene family encodes co-receptors exclusively detected in gamma-delta T cells, which play important roles in immune responses to develop recall responses to antigens.^17^ CD7 is another T-cell receptor transcriptionally upregulated in the CHV group. This protein is expressed in NK cells and mature CD4+ and CD8+ peripheral T cells, playing an essential role in T-cell and T-cell/B-cell interactions during early lymphoid development.^18^ Expression of the gene encoding the chemokine CXCL10 was downregulated in the CHV group. In two other studies that evaluated correlates of protective immune responses to inactivated influenza vaccines with a systems vaccinology approach, levels of serum CXCL10 correlated with good humoral responders,^19,20^ i.e. production of neutralizing antibodies. Two other genes encoding proteins involved in chemotaxis, CXCL12 and CCR10, were upregulated. The CXCL12 chemokine, also known as stromal cell-derived factor 1 (SDF-1), is a strong chemoattractant for lymphocyte migration from the blood stream into sites of inflammation and the efficient activation of these cells under proinflammatory environments.^21^ CCR10 is a chemokine receptor whose ligands, CCL27 and CCL28, are expressed on melanocytes, plasma cells and, of interest for protection against a skin ectoparasite, skin-homing T cells.^22^ Interestingly, previous studies have found a greater accumulation of CD3 + T lymphocytes and WC1 + T lymphocytes in the infested skin of genetically tick-resistant bovines,^23–25^ which also recognize many more tick salivary proteins with IgG antibodies than the tick-susceptible breed employed in the present study.^26^ These results could signify that by neutralizing components of tick saliva with antibodies induced by the multicomponent vaccine, a tissue repair response is able to proceed and hinder tick attachment and blood feeding.

The GSEA, in turn, was enriched for modules reflecting antigen presentation and phosphatidylinositol signaling of immune cells, among others. These modules were also reported in other studies to be enriched by vaccination with antigens from *Plasmodium falciparum*^27^ or by intradermal challenges with sporozoites^28^ of this parasite. In fact, there was great concordance between upregulated and downregulated transcriptional modules between this latter study and the present study. Phosphatidylinositol signaling is also significantly associated with neutralizing antibody titers after measles vaccination.^29^ Finally, another blood transcriptional module enriched in the GSEA of the present study, Mismatch Repair (I) (M22.0 in Fig. 2), was also found to be correlated with serum neutralizing antibody titers in sheep vaccinated with inactivated foot and mouth disease virus.^30^ Of interest for the present study, this pathway is one of the mutagenic DNA damage responses that participate in somatic hypermutation to increase antibody affinity.^31^

In conclusion, all these genes and pathways should play important roles in controlling tick infestation in vaccinated animals with the participation of molecules involved in skin immune responses. Finally, the bovine transcriptome data based on a Systems Vaccinology approach presented herein, will provide findings and insights to help the rational vaccine design for development of protective vaccines for ticks that rely on generating neutralizing antibodies.

## Methods

### Blood sample collection

A total of 24 blood samples were collected from vaccinated and control calves (*n* = 4, each group) at three different time points during experimental vaccination: (a) before vaccination (BV) or administration of control adjuvant (BA); (b) after vaccination (AV) or administration of control adjuvant (AA); and (c) after challenge tick infestation in vaccinated (CHV) and control adjuvant (CHA) animals. Whole peripheral blood was collected from the jugular vein using BD Vacutainer and PAXgene Blood RNA tubes (PreAnalytix, BD) according to the manufacturer’s instructions. The samples were stored at −70 °C until further use. All animals were housed in the same cattle shed and arranged individually in stalls with containment fencing.

### RNA isolation

Whole peripheral blood samples were stabilised in PAXgene Blood RNA tubes, followed by isolation and purification using the PAXgene Blood RNA Kit (PreAnalytix) according to the manufacturer’s instructions. Briefly, the blood samples were defrosted and centrifuged, and subsequently, the supernatants were removed. The pellets containing whole-blood cell lysate were washed and resuspended in the buffer provided in the kit, followed by the binding, DNase I treatment, washing and eluting of total RNA using the column systems and buffers provided in the kit. Total RNA samples were quantified using a NanoDrop 1000 spectrophotometer, and the RNA quality was analysed using a lab-on-chip Agilent 2100 Bioanalyzer. The RNA samples were precipitated and submitted to a sequencing service.

### RNA sequencing

Sequencing data were generated on an Illumina platform at the High-Throughput Sequencing and Genotyping Unit of the Roy J. Carver Biotechnology Center, University of Illinois, Urbana-Champaign, Illinois, USA. All procedures were performed according to the manufacturer’s instructions. The libraries were prepared using a TruSeq RNA Sample Prep kit and sequenced using a TruSeq SBS kit v3-HS on an Illumina HiSeq2000 system. Briefly, after sample quality control analysis, the poly(A) + mRNA samples were purified from total RNA using oligo(dT) magnetic beads, followed by fragmentation, cDNA conversion using random primers, end repair, 3′-end adenylation and adaptor (barcode) ligation. The 24 libraries were combined into six pools, quantified through qPCR, and sequenced in four lanes for 100 cycles. Single-end reads of 100 nucleotides were analysed using Casava 1.8 software (pipeline 1.9) and the de-multiplexed compressed fastq files downloaded from the server of the sequencing service unit.

### RNA-seq data analysis

The quality control of the fastq files containing the raw sequence reads was performed using FastQC (Babraham Institute; available at: http://www.bioinformatics.babraham.ac.uk/projects/fastqc/). No filtering step was performed with the fastq files, as the quality of the results from FastQC reports showed a high level of average quality reads. Next, the reads were aligned to the bovine reference genome (*Bos taurus* genome assembly UMD3.1 downloaded from Ensembl) using TopHat2 mapper, version 2.0.9.^32^ The quantification of mapped reads was performed using HTSeq version 0.5.4,^33^ whose read count outputs were used as inputs for differential expression analysis calculated with edgeR package version 3.2.4^34^ using the generalised linear model (GLM) likelihood ratio test. A threshold of FDR < 0.05 were applied to obtain the differentially expressed genes in all comparisons. Because RNA-seq measures absolute numbers of transcripts and because qRT-PCR correlates poorly with those genes presenting either low or high levels of expression in transcriptomes,^35^ we proceeded to validate the data biologically by seeking functional correlations with the bioinformatic strategies described in the next section, with the relevant responses measured in our previous studies, in particular the study that generated the samples employed herein, as well as with relevant responses measured in studies by other investigators; these correlations will be presented in the Results and Discussion sections. The RPKM (reads per kilobase per million) was calculated to normalise the read counts according to gene length (sum of exons for a transcript) based on edgeR user’s guide instructions. The gene length was obtained using an in-house perl script, using information from the genomic features of Ensembl IDs. For genes with more than one transcript, the larger transcript was considered. Hierarchical clustering (HCL) to examine the gene expression pattern was performed with the RPKM values using a standard statistical algorithm previously described.^36^ HCL employed Euclidean distance for metric calculations, and the average linkage method was used to indicate the cluster-to-cluster distance in the hierarchical trees, which were displayed as heatmaps. HCL analyses were performed using MeV (Multi Experiment Viewer) software.^15^

For functional analyses, the differential expression profiles obtained from all comparisons were functionally enriched using MetaCore™ from Clarivate Analytics, a web-based software for functional analyses based on high-quality, manually curated databases. Pathway and network analyses and Gene Ontology (GO) processes were retrieved using an integrated workflow available in MetaCore™. Because the databases of this software only contain information from humans and rodents, the bovine gene IDs were converted into orthologous human gene IDs using the BioMart database^13^ through the Ensembl website. Gene set enrichment analysis (GSEA) was performed using Blood Transcription Modules (BTMs) as described elsewhere.^14^ Log CPM values were used for comparisons to baseline gene expression. To determine significant associations of BTM with tick challenge phenotypes after adjuvant injection or vaccination, we used baseline-normalized gene expression, as previously described.^37^ Parameters included signal to noise ranking metric and weighted enrichment statistic with 1000 permutations.

### Ethics statement

The calves were maintained according to the guidelines of the Committee for Ethics in Animal Experimentation of the Ribeirão Preto School of Medicine, University of São Paulo (CETEA-FMRP/USP, certificate numbers 055/2007, 210/2008 and 102/2009).

### Reporting summary

Further information on research design is available in the Nature Research Reporting Summary linked to this article.

## Supplementary information

Caption for Supplementary Data 1

Supplementary Data 1

Reporting Summary

## Data Availability

RNA-seq data are deposited in the ArrayExpress/EMBL-EBI database under accession number E-MTAB-8022.
