# Supplementary material for: Blood transcriptome profile induced by an efficacious vaccine formulated with salivary antigens from cattle ticks
Source: NPJ Vaccines. 2019 Dec 18;4:53. doi: 10.1038/s41541-019-0145-1 (PMC6920353; doi:10.1038/s41541-019-0145-1)
Supplement: Supplementary file 1 — Caption for Supplementary Data 1 [file 41541_2019_145_MOESM1_ESM.pdf]

Supplementary Data 1. List of DEGs in all comparisons (sheets a-d) and list of genes in blood transcriptional modules (BTMs; sheet e).
