# Supplementary material for: Blood transcriptome profile induced by an efficacious vaccine formulated with salivary antigens from cattle ticks
Source: NPJ Vaccines. 2019 Dec 18;4:53. doi: 10.1038/s41541-019-0145-1 (PMC6920353; doi:10.1038/s41541-019-0145-1)
Supplement: Supplementary file 3 — Reporting Summary [file 41541_2019_145_MOESM3_ESM.pdf]

nature research

Corresponding author(s): NRJ Vaccines 00400R

Reporting Summary

Nature Research wishes to improve the reproducibility of the work that we publish. This form provides structure for consistency and transparency in reporting. For further information on Nature Research policies, see Authors & Referees and the Editorial Policy Checklist.

Statistical parameters

When statistical analyses are reported, confirm that the following items are present in the relevant location (e.g. figure legend, table legend, main text, or Methods section).

n/a	Confirmed	
☐	☒	The exact sample size (*n*) for each experimental group/condition, given as a discrete number and unit of measurement
☐	☒	An indication of whether measurements were taken from distinct samples or whether the same sample was measured repeatedly
☐	☒	The statistical test(s) used AND whether they are one- or two-sided<br>*Only common tests should be described solely by name; describe more complex techniques in the Methods section.*
☒	☐	A description of all covariates tested
☒	☐	A description of any assumptions or corrections, such as tests of normality and adjustment for multiple comparisons
☒	☐	A full description of the statistics including central tendency (e.g. means) or other basic estimates (e.g. regression coefficient) AND variation (e.g. standard deviation) or associated estimates of uncertainty (e.g. confidence intervals)
☒	☐	For null hypothesis testing, the test statistic (e.g. *F*, *t*, *r*) with confidence intervals, effect sizes, degrees of freedom and *P* value noted<br>*Give P values as exact values whenever suitable.*
☒	☐	For Bayesian analysis, information on the choice of priors and Markov chain Monte Carlo settings
☒	☐	For hierarchical and complex designs, identification of the appropriate level for tests and full reporting of outcomes
☒	☐	Estimates of effect sizes (e.g. Cohen's *d*, Pearson's *r*), indicating how they were calculated
☒	☐	Clearly defined error bars<br>*State explicitly what error bars represent (e.g. SD, SE, CI)*

Our web collection on statistics for biologists may be useful.

Software and code

Policy information about availability of computer code

Data collection	[illegible]
Data analysis	[illegible]

For manuscripts utilizing custom algorithms or software that are central to the research but not yet described in published literature, software must be made available to editors/reviewers upon request. We strongly encourage code deposition in a community repository (e.g. GitHub). See the Nature Research guidelines for submitting code & software for further information.

Data

Policy information about availability of data

All manuscripts must include a data availability statement. This statement should provide the following information, where applicable:

- Accession codes, unique identifiers, or web links for publicly available datasets
- A list of figures that have associated raw data
- A description of any restrictions on data availability

[illegible]

Field-specific reporting

Please select the best fit for your research. If you are not sure, read the appropriate sections before making your selection.

☐ Life sciences ☐ Behavioural & social sciences

For a reference copy of the document with all sections, see nature.com/authors/policies/ReportingSummary-flat.pdf

Life sciences

Study design

All studies must disclose on these points even when the disclosure is negative.

Sample size	Vaccine trials with large outbred animals employ an equivalent number of animals / experimental group.
Data exclusions	No data were excluded
Replication	We employed four outbred animals / experimental group
Randomization	We employed unrelated animals with known pedigrees
Blinding	Investigators were blinded regarding treatments (vaccine or adjuvant)

Materials & experimental systems

Policy information about availability of materials

n/a	Involved in the study
☐	☒ Unique materials
☒	☐ Antibodies
☒	☐ Eukaryotic cell lines
☐	☒ Research animals
☒	☐ Human research participants

Unique materials

Obtaining unique materials	Antigens of vaccine are patented.

Antibodies

Antibodies used	
Validation	

Eukaryotic cell lines

Policy information about cell lines

Cell line source(s)	
Authentication	
Mycoplasma contamination	
Commonly misidentified lines (See ICLAC register)	

Research animals

Policy information about studies involving animals; ARRIVE guidelines recommended for reporting animal research

Animals/animal-derived materials: Bos taurus taurus; Holstein-Friesian; female; 4-7 months; known pedigree

Human research participants

Policy information about studies involving human research participants

Population characteristics

Method-specific reporting

n/a	Involved in the study
☒	☐ ChIP-seq
☒	☐ Flow cytometry
☒	☐ Magnetic resonance imaging

ChIP-seq

Data deposition

- ☐ Confirm that both raw and final processed data have been deposited in a public database such as GEO.
- ☐ Confirm that you have deposited or provided access to graph files (e.g. BED files) for the called peaks.

Data access links
May remain private before publication.

Files in database submission

Genome browser session
(e.g. UCSC)

Methodology

Replicates

Sequencing depth

Antibodies

Peak calling parameters

Data quality

Software

Flow Cytometry

Plots

Confirm that:

- ☐ The axis labels state the marker and fluorochrome used (e.g. CD4-FITC).
- ☐ The axis scales are clearly visible. Include numbers along axes only for bottom left plot of group (a 'group' is an analysis of identical markers).
- ☐ All plots are contour plots with outliers or pseudocolor plots.
- ☐ A numerical value for number of cells or percentage (with statistics) is provided.

Methodology

Sample preparation

Instrument

Software

Cell population abundance

Gating strategy

☐ Tick this box to confirm that a figure exemplifying the gating strategy is provided in the Supplementary Information.

Magnetic resonance imaging

Experimental design

Design type

Design specifications

Behavioral performance measures

Acquisition

Imaging type(s)

Field strength

Sequence & imaging parameters

Area of acquisition

Diffusion MRI ☐ Used ☐ Not used

Preprocessing

Preprocessing software

Normalization

Normalization template

Noise and artifact removal

Volume censoring

Statistical modeling & inference

Model type and settings

Effect(s) tested

Specify type of analysis: ☐ Whole brain ☐ ROI-based ☐ Both

Statistic type for inference
(See Eklund et al. 2016)

Correction

Models & analysis

n/a	Involved in the study
☐	☐ Functional and/or effective connectivity
☐	☐ Graph analysis
☐	☐ Multivariate modeling or predictive analysis

Functional and/or effective connectivity [illegible]

Graph analysis [illegible]

Multivariate modeling and predictive analysis [illegible]

Behavioural & social sciences

Study design

All studies must disclose on these points even when the disclosure is negative.

Study description [illegible]

Research sample [illegible]

Sampling strategy [illegible]

Data collection [illegible]

Timing [illegible]

Data exclusions [illegible]

Non-participation [illegible]

Randomization [illegible]
